# Charge-Transfer Complexes in Organic Field-Effect Transistors: Superior Suitability for Surface Doping

**DOI:** 10.1021/acsami.2c09168

**Published:** 2022-09-20

**Authors:** Adara Babuji, Alba Cazorla, Eduardo Solano, Carsten Habenicht, Hans Kleemann, Carmen Ocal, Karl Leo, Esther Barrena

**Affiliations:** † Institut de Ciència de Materials de Barcelona (ICMAB), Campus de la UAB, Bellaterra, Barcelona 08193, Spain; ‡ Dresden Integrated Center for Applied Physics and Photonic Materials (IAPP), Dresden 01062, Germany; § NCD-SWEET beamline, ALBA Synchrotron Light Source, C/ de la Llum 2-26. Cerdanyola del Vallès, Barcelona 08290, Spain

**Keywords:** organic semiconductor, doping, charge-transfer complexes, cocrystals, OFETs

## Abstract

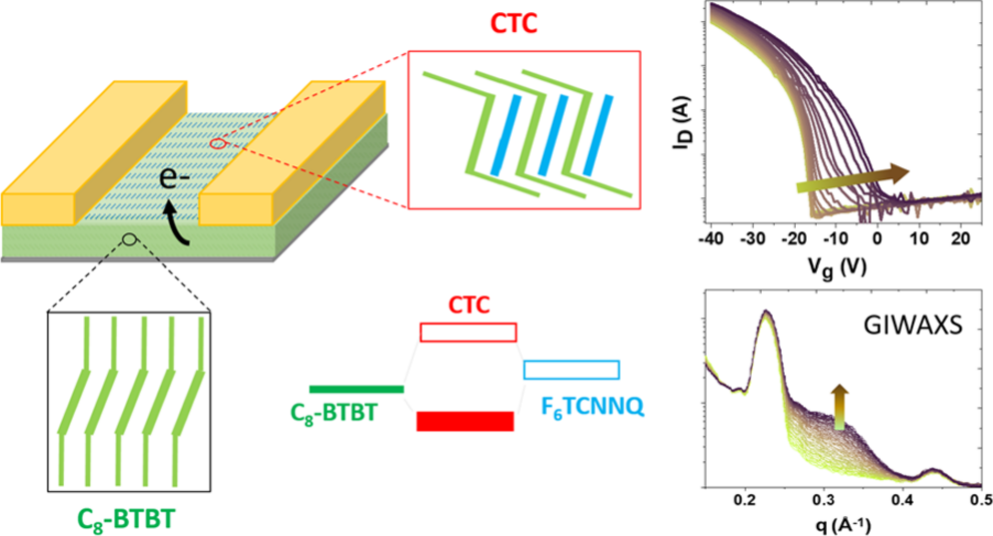

We demonstrate the key role of charge-transfer complexes in surface doping as a successful methodology for improving channel field-effect mobility and reducing the threshold voltage in organic field-effect transistors (OFETs), as well as raising the film conductivity. Demonstrated here for 2,7-dioctyl[1]benzothieno[3,2-*b*][1]benzothiophene (C_8_-BTBT) doped with 2,2′-(perfluoronaphthalene-2,6-diylidene)dimalononitrile (F_6_TCNNQ), channel doping by sequential deposition is consistently rationalized by the development of a cocrystalline structure that forms and evolves from the surface of the organic semiconductor film without trading the thin-film structure integrity. This scenario brings higher benefits for the device operation than doping by codeposition, where a decrease in the field-effect mobility of the device, even for a dopant content of only 1 mol %, makes codeposition less suitable. Insight into the structural and electronic properties of the interface satisfactorily explains the improved performance of OFETs upon the incorporation of the dopant and provides an understanding of the mechanism of doping in this system.

## Introduction

Doping organic semiconductor (OSC) materials remains a topic of scientific and technological interest for device optimization. The most successful application of molecular doping is found in organic light-emitting diodes (OLEDs) and already materialized in the technology of OLED displays. The incorporation of molecular dopants is indispensable to obtain n-type or p-type charge-transport layers (for electrons or holes, respectively), a strategy that has been extended with great success also to organic photovoltaic (OPV) devices. In the case of organic field-effect transistors (OFETs), although the use of dopants is still relatively limited, there is an increasing interest in exploring doping as an important design parameter to fill unwanted trap states,^1,2^ improve device stability,^3^ tune the threshold voltage (*V*_th_), and have control over the majority charge carrier type.^4−6^ Unlike OLEDs and OPV devices, in OFETs, the field-effect charge carrier mobility (μ) is the key figure of merit, which crucially depends on the overall structural quality of the OSC film. The most common strategy to increase μ is the addition of molecular dopants to the OSC film by codeposition of both materials (also referred to as bulk doping). The downside of bulk doping of OFETs based on highly ordered OSC films is that it may produce structural disorder and the adverse consequent decrease of μ.^7^ In this context, the sequential deposition of the OSC and the dopant has been introduced in OFETs. The standard approach consists of adding a dopant layer solely under the top electrodes for reducing contact resistance. The so-called contact doping increases the carrier concentration at the contact region and aids in reducing the depletion layer, filling interfacial traps, and improving the charge transport across the OSC film (reducing the access resistance) in a staggered bottom gate geometry.^8−10^ The deposition of a thin dopant layer onto the OSC film in the channel has attracted interest as an additional approach to improve the device operation by achieving enhanced mobility and reducing threshold voltage by filling trap states in the channel.^3,11−19^ Contact and channel doping, generally referred to as surface doping, relies on integer electron charge transfer between the OSC and the molecular dopant (i.e., ion pair formation).

In the past years, a wealth of studies have reported that bulk doping may occur via different physical mechanisms. In particular, for planar small-molecule OSCs such as 2,3,5,6tetrafluoro-7,7,8,8-tetracyanoquinodimethane (F_4_TCNQ) or 2,2′-(perfluoronaphthalene-2,6-diylidene)dimalononitrile (F_6_TCNNQ) as p-type dopants, doping has been proposed to be dominated by the formation of a ground-state charge-transfer complex (CTC) due to the interaction between the π systems of the dopant and OSC molecules. Their Frontier molecular orbitals hybridize, forming a bonding and an antibonding supramolecular orbital, thus exhibiting a new set of energy absorption features within the optical gap of the pristine materials.^20−24^ CTC formation is mostly detected by UV–vis/NIR spectroscopy, although for some OSCs, a structural identification has been made, giving evidence of the formation of CTC mixed crystals. For example, for the admixture of 2,7-didecyl[1]benzothieno[3,2-*b*][1]benzothiophene (C_10_-BTBT) and F_4_TCNQ, the CTC arises from a mixed 1:1 structure of cofacially stacked OSC/dopant molecules.^25^ Within this scenario, the CTC cocrystals inside the pristine C_10_-BTBT film were proposed to act as p-type dopants. The conductivity was observed to increase with the dopant ratio, achieving a maximum value for a dopant concentration of 5 mol % and decreasing beyond this value. Other articles^22,26^ have reported differences in the dependence of the conductivity on dopant concentration.^5,7,27^ Although current studies highlight the potential offered by CTC for electrical doping in small-molecule OSCs, they also reveal the difficulty in tailoring the microstructure resulting from mixing the two molecular materials, that is, the fraction, order, and spatial distribution of the cocrystalline regions within the OSC film. The efficiency of CTC for doping is still under debate.

In this work, we demonstrate that surface doping may take place via CTC and we explore its key role in improving the channel field-effect mobility and reducing the threshold voltage (*V*_th_) in OFETs, as well as the suitability for raising the film’s conductivity. This strategy, demonstrated here for C_8_-BTBT OFETs doped with F_6_TCNNQ, proves to bring higher benefits for the device operation than codeposition, which conversely causes a decrease in the device field-effect mobility, even for a dopant content of only 1 mol %. Doping is consistently explained for both sequential and coevaporation deposition by the development of a C_8_-BTBT/F_6_TCNNQ cocrystalline structure with CTC properties. We provide a detailed description of the interface as well as a correlation between the electrical and structural properties by a combination of *in situ* synchrotron grazing-incidence wide-angle X-ray scattering (GIWAXS), ultraviolet–visible absorption (UV–vis), ultraviolet photoelectron spectroscopy (UPS), Kelvin probe force microscopy (KPFM), and transport measurements in OFETs.

## Experimental Methods

### Materials and Thin Film Deposition

C_8_-BTBT was purchased from Sigma-Aldrich and F_6_TCNNQ from Novaled AG Dresden. Si(100) substrates with 100 nm-thick SiO_2_ were used to measure GIWAXS and atomic force microscopy (AFM) and a glass substrate was used to measure UV–vis absorption. Both substrates were cleaned before molecule deposition by sonication in acetone and ethanol for 10 min, respectively. C_8_-BTBT and F_6_TCNNQ molecules were thermally evaporated under vacuum (∼10^–8^ mbar) at room temperature on cleaned substrates.

### OFET Fabrication

Staggered bottom-gate OFETs were fabricated on 100 nm-thick SiO_2_ substrates. To passivate the substrate, a 40 nm-thick CYTOP [CYTOP-809M diluted in CTL 180 (2:7)] layer was spin-coated on top, which makes the effective dielectric to be the sum of the 100 nm-thick SiO_2_ and the 40 nm-thick CYTOP layer. Afterward, organic layers were deposited, and the top 50 nm-thick Au contacts (source and drain electrodes) were also deposited on top using a shadow mask. There were three sets of OFETs with five different channel lengths (*L* = 80, 130, 180, 230, and 330 μm), all with a channel length of *W* = 2 mm. The nominal amount of each deposited material was measured by a dedicated quartz microbalance (QM). For coevaporated films, the percentage of the dopant is given in molar mass, whereas in samples prepared by sequential deposition of F_6_TCNNQ on C_8_-BTBT, the amount of the dopant is given in Angstroms. Each device was isolated from the others by scratching out the OSC film between them. Characterization of OFETs was done using a semiconductor characterization system (SCS Parameter Analyzer) with a probe station. *V*_on_ is the voltage at which the drain current starts to depend on the gate voltage.

Transfer characteristics were measured in the linear and saturation regime, swept forward and reverse. The turn-on voltage has been calculated from the log *I*_D_*versus V*_g_ plot as an intercept with the horizontal line defined by the off-state current. The mobility and the threshold voltage (*V*_th_) were extracted for linear regime according to
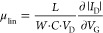
1
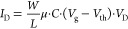
2where *C* is the insulator capacitance per unit area and *W* and *L* are the width and length of the channel, respectively. The total capacitance of the CYTOP coated Si/SiO_2_ substrate is 19.83 nF·cm^–2^, calculated by 1/*C*_total_ = (1/*C*_CYTOP_ + 1/*C*_SiO_2__), where *C*_SiO_2__ is 34.6 nF·cm^–2^ and *C*_CYTOP_ has been obtained from the layer thickness (*d* = 40 nm) *C*_CYTOP_ = ε_0_·ε/*d* (with ε = 2.1).

Coplanar bottom-gate OFETs were fabricated on 200 nm-thick SiO_2_ substrates. 50 nm-thick Au electrodes were deposited on top of the cleaned SiO_2_ using a shadow mask for a 400 μm channel length. To improve the contacts, prior to metal deposition, a self-assembled monolayer was formed on SiO_2_ by immersion in a 1 mM perfluorodecanethiol ethanol solution. Electrode wiring was attached with silver paste and channeled out of the vacuum deposition chamber via low-current feedthrough. The OFET characteristics were measured for the *in situ*-grown 15 nm-thick C_8_-BTBT film and during the subsequent deposition of F_6_TCNNQ on its top. All transistor parameters are extracted from the linear regime of operation.

### Grazing-Incidence Wide-Angle X-ray Scattering

Experiments of GIWAXS were performed at the BL11-NCD-SWEET beamline of the ALBA Synchrotron (Spain) using a photon energy of *E* = 12.4 keV. The incident angle was varied between 0.09 and 0.15°. A large-area 2D Rayonix LX 255-HS detector was used to capture 2D images of the diffraction patterns, which consists of a pixel array of 2880 × 960 (*V* × *H*) with a pixel size of 88.54 × 88.54 μm^2^ for the binning employed. The scattering vector *q⃗* was calibrated using Cr_2_O_3_ as a calibration standard. For *in situ* measurements, diffraction patterns were recorded for every 0.4 Å of F_6_TCNNQ until a coverage of 35 Å. For larger coverages, five images (exposure time of 0.2 s) were collected every 30 s and summed up to improve the signal-to-noise ratio. Because of the grazing-incidence scattering geometry employed, cuts along the *Z*-axis on the area detector are not true specular scans. The data are converted to reciprocal space maps with the *q*_*z*_ and in-plane (*q*_*xy*_) components of the scattering vector corresponding to the directions perpendicular and parallel to the surface, respectively. Real space distances (*d*) are obtained from the modulus of the scattering vector (*q*) using *d* = 2π/*q*. For the GIWAXS measurements performed *in situ* during the deposition of F_6_TCNNQ, we used a portable ultrahigh vacuum (UHV) chamber with a 360° beryllium window (transparent to X-ray), equipped with a QM and effusion cells for molecular evaporation. First, a 30 nm-thick C_8_-BTBT film was grown on CYTOP (40 nm)/SiO_2_ (100 nm) and GIWAXS 2D maps were recorded *in situ* during the evaporation of F_6_TCNNQ. The evaporation was interrupted at certain coverages to perform a set of measurements at different incident angles. For each angle, the sample was moved stepwise to a fresh spot, avoiding radiation damage issues. Changing the incident angle, the sampled depth was changed from the topmost surface for 0.09° to the entire thickness for 0.15°. Films prepared by coevaporation were measured *ex situ* and in ambient conditions for the same incidence angles.

### Atomic Force Microscopy

AFM was performed using a commercial head and electronics from Nanotec Electronica. Surface potential maps were acquired by frequency-modulated KPFM in single-pass mode (bias voltage applied to the tip). All AFM data presented here were analyzed by using the WSxM freeware.^28^

### UV–Vis Absorption Spectroscopy

After sample preparation in UHV, the ultraviolet–visible absorption measurements were performed in ambient conditions using a JASCO V780 UV–vis–NIR spectrophotometer. Twin samples of those used for GIWAXS on glass substrates were used for these measurements.

### Ultraviolet Photoelectron Spectroscopy

Molecules were evaporated on the Si substrate in UHV and transferred to another chamber for UPS characterization without breaking the vacuum. The UPS spectra were acquired using a PHOIBOS 100 analyzer system (Specs) and a helium discharge lamp (He I, 21.22 eV). The base pressure was 10^–10^ mbar. The energy resolution was determined to be 150 meV based on the width of the Fermi edge of a silver substrate.

## Results and Discussion

### Bulk Doping

**Figure 1:**
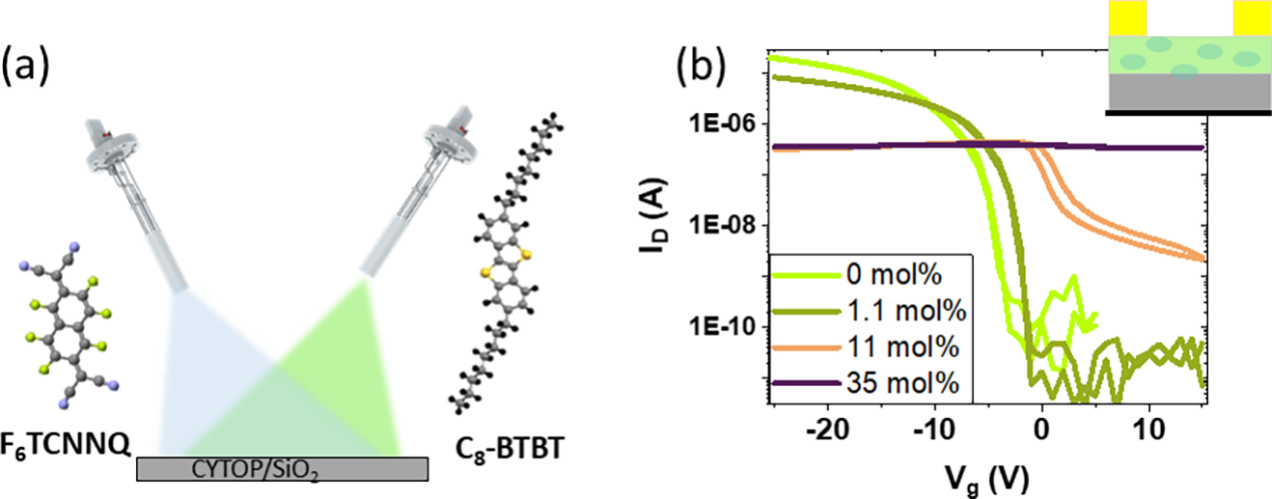
(a) Cartoon representing the coevaporation of C_8_-BTBT and F_6_TCNNQ. The structures of each molecule are shown. (b) Linear transfer curves at *V*_D_ = −5 V for OFETs with staggered bottom-gate geometry for coevaporated thin films with the indicated concentrations of F_6_TCNNQ (channel length = 230 μm).

We focus first on the coevaporation of F_6_TCNNQ and C_8_-BTBT (Figure 1a), which is expected to produce homogeneous and reproducible films with the desired OSC/dopant ratio by choosing the appropriate sublimation rates. The electrical properties have been evaluated in OFETs fabricated with the staggered bottom-gate architecture for various F_6_TCNNQ concentrations. One set of 15 devices was fabricated for each OSC/dopant ratio using 40 nm CYTOP/100 nm SiO_2_ as a substrate (see Experimental Methods). Figure 1b shows representative transfer characteristics of OFETs operating in a linear regime, with F_6_TCNNQ concentrations up to 35 mol %. The respectively extracted parameters are listed in Table 1 for devices with *L* = 230 μm. The output curves and results for other channel lengths are given in Figure S1.

**Table 1:** OFET Parameters Extracted from the Transfer Curves of Codeposited Thin-Film OFETs (Linear Operation Regime) with Diverse Concentrations of F_6_TCNNQ with Respect to C_8_-BTBT and a Channel Length of 230 μm

F_6_TCNNQ (mol %)	*V*_on_ (V)	*I*_on_/*I*_off_	*V*_th_ (V)	μ (cm^2^/Vs)	subthreshold swing (V/dec)
0	–3 ± 0.7	(1.13 ± 1.03)·10^6^	–(3 ± 0.5)	1.35 ± 0.05	0.55 ± 0.47
1.1	–0.33 ± 0.58	(7.7 ± 10)·10^6^	–(1.07 ± 0.89)	0.31 ± 0.23	0.93 ± 0.21
11	4.33 ± 0.58	(7.9 ± 4.9)·10^1^	4.7 ± 0.4		3.26 ± 0.09
35	conductive films (cannot switch off)				

For the device with the smallest concentration of F_6_TCNNQ (1.1 mol %), a small shift in the threshold voltage (*V*_th_) toward zero is observed. Such a shift in *V*_th_ can be caused by the generation of free charge carriers but also by the filling of traps (either at the interface between the gate insulator and OSC or within the OSC) as already reported for low doping concentrations.^1,29,30^ A dopant concentration of 11 mol % results in a larger *V*_th_ shift toward a more positive value as well as an increase of 2 orders of magnitude (from 10^–10^ to 10^–8^ A) in the off-current (*I*_off_), indicating a provision of free charges in the OFET channel. It is striking that the on-current (*I*_on_) does not increase with the gate voltage (*V*_g_), which prevents a reliable evaluation of the field-effect mobility. The fact that the current is smaller than in 1.1 mol %-doped films is an indication that the mobility has been reduced. Notably, when the dopant concentration reaches 35 mol %, deterioration of the device operation leads to an increase in the electrical conductivity. As it can be seen in Table 1, F_6_TCNNQ bulk doping of C_8_-BTBT has a detrimental impact on the field-effect mobility that worsens with increasing concentration. Deterioration of the field-effect mobility upon bulk doping is a common observation for small-molecule OSCs as a result of the increased structural disorder.^31^

**Figure 2:**
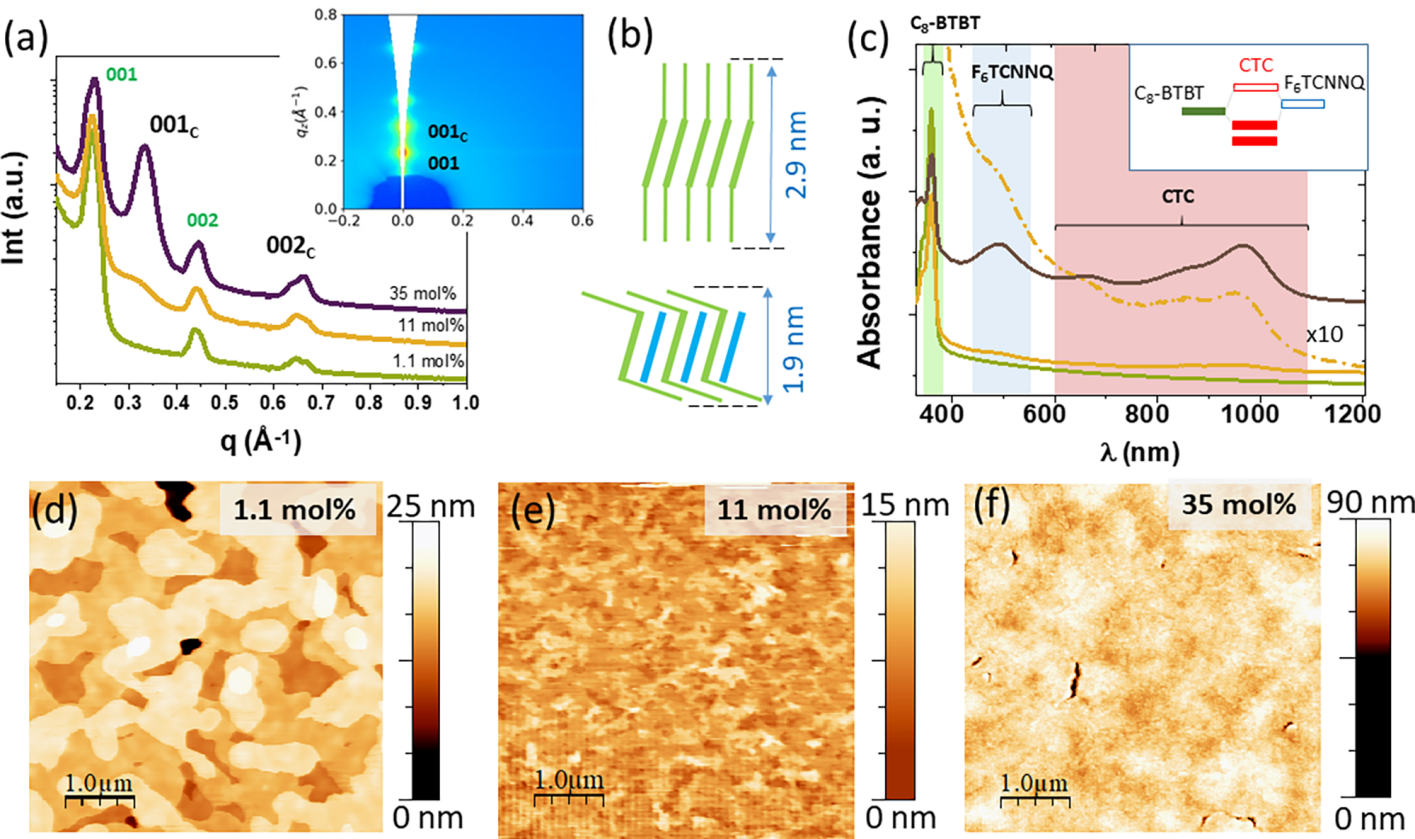
(a) OOP section cuts obtained from 2D GIWAXS images for the diverse coevaporated thin films of C_8_-BTBT and F_6_TCNNQ on CYTOP/SiO_2_ with the indicated F_6_TCNNQ concentrations. (001) and (002) labels correspond to C_8_-BTBT, while (001)_C_ and (002)_C_ arise from the formation of cocrystals. Inset: GIWAXS pattern (*q*_*z*_–*q*_*xy*_) for 35 mol %. (b) Cartoons of the respective C_8_-BTBT (top) and 1:1 cocrystal (bottom) packing. (c) UV–vis absorption spectra for the same three F_6_TCNNQ concentrations. The dashed–dotted curve is 10 times the curve for 11 mol %. (d–f) Topographic AFM images acquired at the channel region of the corresponding OFETs.

The structure of the thin films has been characterized by GIWAXS on a similar set of codeposited films on glass substrates. The measurements were acquired at an incident angle of 0.15°, above the critical angle of the thin film but below the critical angle of the substrate, ensuring the X-ray beam penetration into the thin film but not into the support (see Experimental Methods). Figure 2a shows a section cut along the *z*-direction of the detector, hereafter referred to as an out-of-plane (OOP) section cut (see Experimental Methods). The 2D GIWAXS maps are provided in Figure S2. For the whole range of dopant concentrations, the (001) and (002) Bragg peaks from C_8_-BTBT are visible, with *q*_001_ = 0.21 Å^–1^ corresponding to the reported thin-film structure with an interlayer spacing of ∼2.9 nm, coinciding with a nearly upright configuration of the BTBT core and the alkyl chains of the OSC (Figure 2b).^32^ The most significant observation is, however, the emergence of a new peak at *q*_*z*_ = 0.33 Å^–1^ for 11 mol % concentration that becomes prominent for the largest dopant ratio studied and corresponds to an associated interlayer spacing of ∼1.90 nm. This peak is absent in the single-component films of either C_8_-BTBT or F_6_TCNNQ.^26,33^ In analogy to the cocrystal structure reported for C_8_-BTBT-F_4_TCNQ^34,35^ and C_10_-BTBT/F_4_TCNQ,^25^ the new peak is labeled (001)_C_, being attributed to the formation of cocrystals consisting of an alternated stacking C_8_-BTBT-F_6_TCNNQ. The 1:1 mixed structure is driven by the interaction of the respective conjugated cores of the acceptor and OSC. The resulting molecular assembly consists of two-dimensional layers with a mixed π stacking in the plane and vertically separated by the tilted alkyl chains of the C_8_-BTBT molecules. The pronounced molecular tilt leads to a smaller interlayer spacing than for the pure OSC thin films, as schematically illustrated in Figure 2b. The 2D GIWAXS map for the coevaporated film with 35 mol % is shown in the inset of Figure 2a. The observation of (00l) Bragg peaks along *q*_*z*_ for both, C_8_-BTBT and mixed cocrystals, indicates a pronounced (001) fiber texture, that is, with the (001) crystalline plane parallel to the surface. Owing to the overlap and hybridization of molecular orbitals in the 1:1 mixed structure, a CTC is expected to be formed. To verify it, we performed UV–vis absorption spectroscopy on the same samples (Figure 2c). Absorption regions of reported peaks for C_8_-BTBT (360 nm) and F_6_TCNNQ (468 and 517 nm) are highlighted in the plot by green and blue colors, respectively, and coincide with the location of the spectral features obtained for the pristine films of both molecules.^31,36^ A set of new peaks around 650 and 963 nm (a pink region in the plot) emerge at a concentration of 11 mol % and are clearly visible for the 35 mol % film. The absorption band characteristic of F_6_TCNNQ is only unambiguously observed for the highest dopant ratio. The absence of the F_6_TCNNQ^–^ anion peak (that would show up at ∼1140 nm)^37^ rules out integer charge transfer. The observed subgap absorption bands can be attributed to the transitions between the highest occupied molecular orbitals (HOMO and HOMO – 1) of the CTC to its lowest unoccupied molecular orbital (LUMO) (inset in Figure 2c). The experimentally determined optical gap estimated from the lowest energy absorption peak is 1.28 eV, with the HOMO–LUMO transition being higher in energy by 0.56 eV. Similar values were reported for C_10_BTBT-F_6_TCNNQ CTCs.^25^

GIWAXS and UV–vis absorption experiments consistently show that coevaporation of C_8_-BTBT and F_6_TCNNQ leads to the formation of 1:1 cocrystalline regions inside the film of pure C_8_-BTBT. These cocrystals are at the origin of the electrical doping of the OFETs. This strategy has, however, a detrimental effect on field-effect mobility and already manifested itself at a relatively low dopant concentration of 1.1 mol %. Although for all doping ratios, C_8_-BTBT is found to exhibit high lamellar ordering, we presume that the generation of embedded cocrystallites surely introduces structural disorder, altering the size and crystalline ordering of the in-plane domains, with a negative impact on the in-plane transport. This hypothesis is supported by the inspection of the morphology of the OFET channels (Figure 2d–f). The topographic images of 1.1 mol % doped films are similar to those reported for pristine C_8_-BTBT films,^3^ with terraces separated by the expected height of molecules standing up (multiples of ∼3 nm). For a F_6_TCNNQ concentration of 11 mol %, a terraced topography is still visible but exhibits irregular step edges and a considerably reduced lateral size of the terraces. For a concentration of 35 mol %, the topmost surface is featureless except for a few cracks. The observed differences in the morphology point to an explicit decrease in the lateral size of the C_8_-BTBT domains as the most plausible origin of the reduction in field-effect mobility of the OFETs as the dopant/OSC ratio increases.

### Surface Doping

**Figure 3:**
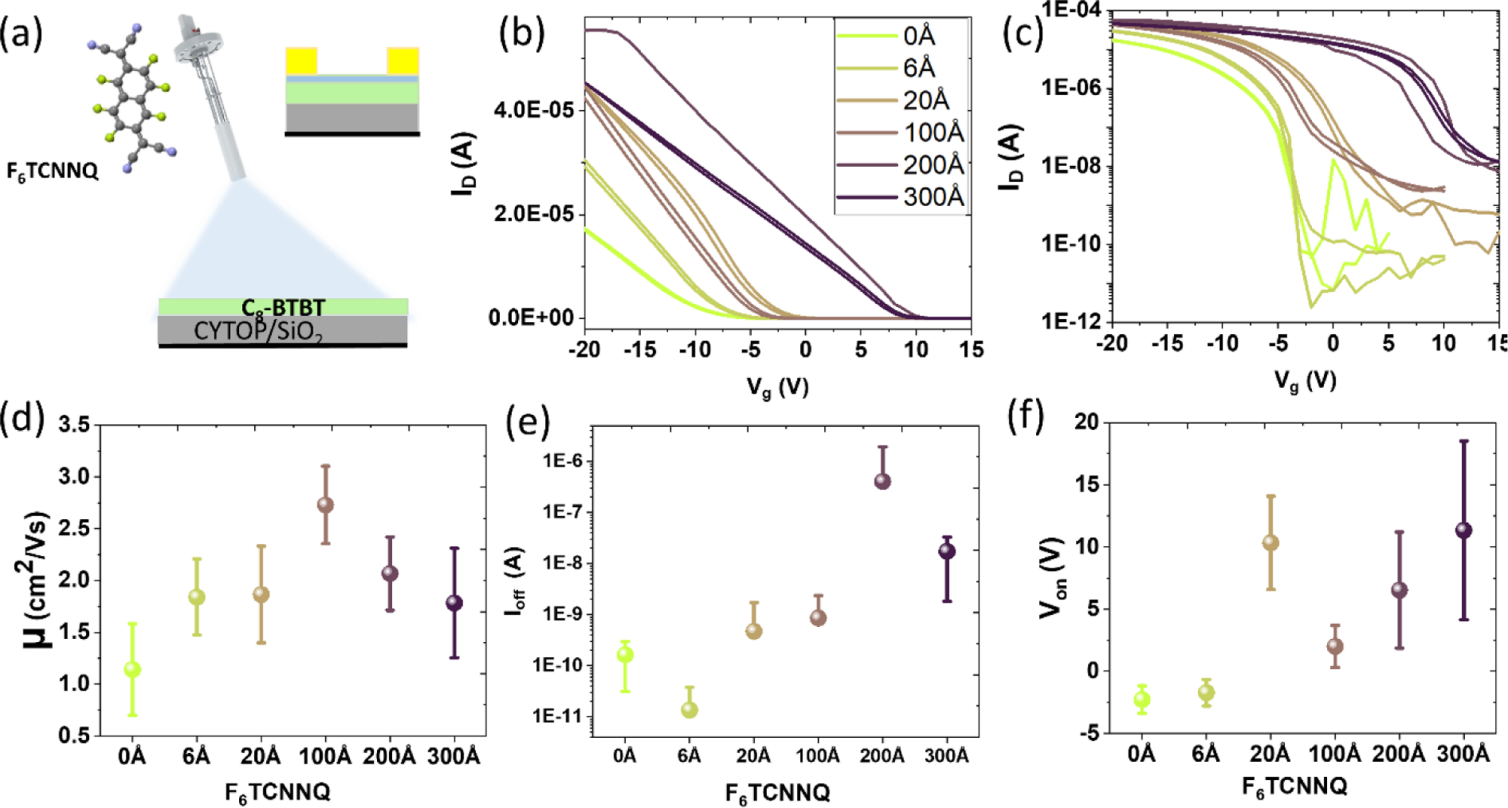
(a) Cartoon representing the *in situ* deposition of F_6_TCNNQ on C_8_-BTBT films. The inset shows the staggered bottom-gate geometry of the fabricated OFETs. The transfer curves at *V*_D_ = −5 V for OFETs with *L* = 180 μm are shown in linear (b) and logarithmic (c) scales. Plots in (d–f) display the effective linear field-effect mobility, drain current when the transistor is off (*I*_off_), and then turn-on voltage (*V*_on_), respectively, extracted from the transfer curves for each F_6_TCNNQ coverage. The depicted data points stand for the averaged values obtained for 15 devices in each case, the error bars corresponding to the standard deviation. The colors in all graphs correspond to OFETs with the F_6_TCNNQ coverages indicated in (b) deposited onto 35 nm-thick C_8_-BTBT films before the Au electrode deposition.

In order to explore surface doping as a compelling alternative to consider, we turn next the focus to the sequential deposition of C_8_-BTBT and F_6_TCNNQ. Surface transfer doping relies on extra carriers (holes or electrons for p-type or n-type doping, respectively) induced by charge transfer at the OSC interface. As mentioned in the Introduction, it has been employed for selective doping of the interface under the electrodes (contact doping) to reduce the contact resistance in staggered bottom-gate OFETs. In addition, surface doping has been employed as well to dope the OFET channel and has been proven to be a suitable strategy for enhancing the morphological and electrical stability of the devices.^3,15,38,39^ Different coverages of F_6_TCNNQ (given in Å, see Experimental Methods) have been deposited on top of a C_8_-BTBT film (Figure 3a). The corresponding OFETs were fabricated with the same architecture and same gate insulator as the codeposited OFETs.

Representative transfer curves under linear regime operation (*V*_D_ = −5 V) for fabricated OFETs (*L* = 180 μm) with coverages from 0 to 300 Å of F_6_TCNNQ on the surface of 35 nm-thick C_8_-BTBT films are shown in Figure 3b,c, in linear and logarithmic scales, respectively. The corresponding output curves are provided in Figure S3. One set of 15 devices for each coverage was analyzed to extract the effective field-effect mobility, the drain current for off-state (*I*_off_), and the turn-on voltage (*V*_on_) depicted in Figure 3d–f, respectively. The trends observed with increasing dopant coverage, that is, a considerable shift of threshold voltage (from −2.5 to 12 V) toward more positive voltages and an increase in the off-current, are associated with an increase in the density of free carriers due to p-type doping. Importantly, unlike the case of bulk-doped OFETs by coevaporation, the mobility of holes in C_8_-BTBT presented in Figure 3d does not decrease with the amount of dopant. On the contrary, μ increases and, for a F_6_TCNNQ coverage of 100 Å (equivalent to a dopant ratio of about 40 mol %), it acquires a maximum value of more than twice its value for the pristine device. Beyond this optimum coverage, the mobility decreases. Nevertheless, the generation of free carriers by doping further increases, as evidenced by the increase in the *I*_off_ and the progressive shift of *V*_on_ toward positive values. A systematic analysis of μ as a function of the dopant coverage is provided in Figure S4 for a large number of OFETs with channel lengths ranging from *L* = 80 μm to *L* = 330 μm. This extensive survey points to the same trend observed in Figure 3d, indicating the robustness of the results. Further results (provided in Figure S5) show that for low dopant coverages, the mobility is identically enhanced regardless of whether the dopant is below the contacts or only in the channel. Consequently, the improvement of field-effect mobility can be considered as one of the intrinsic benefits of induced surface doping at the channel.

**Figure 4:**
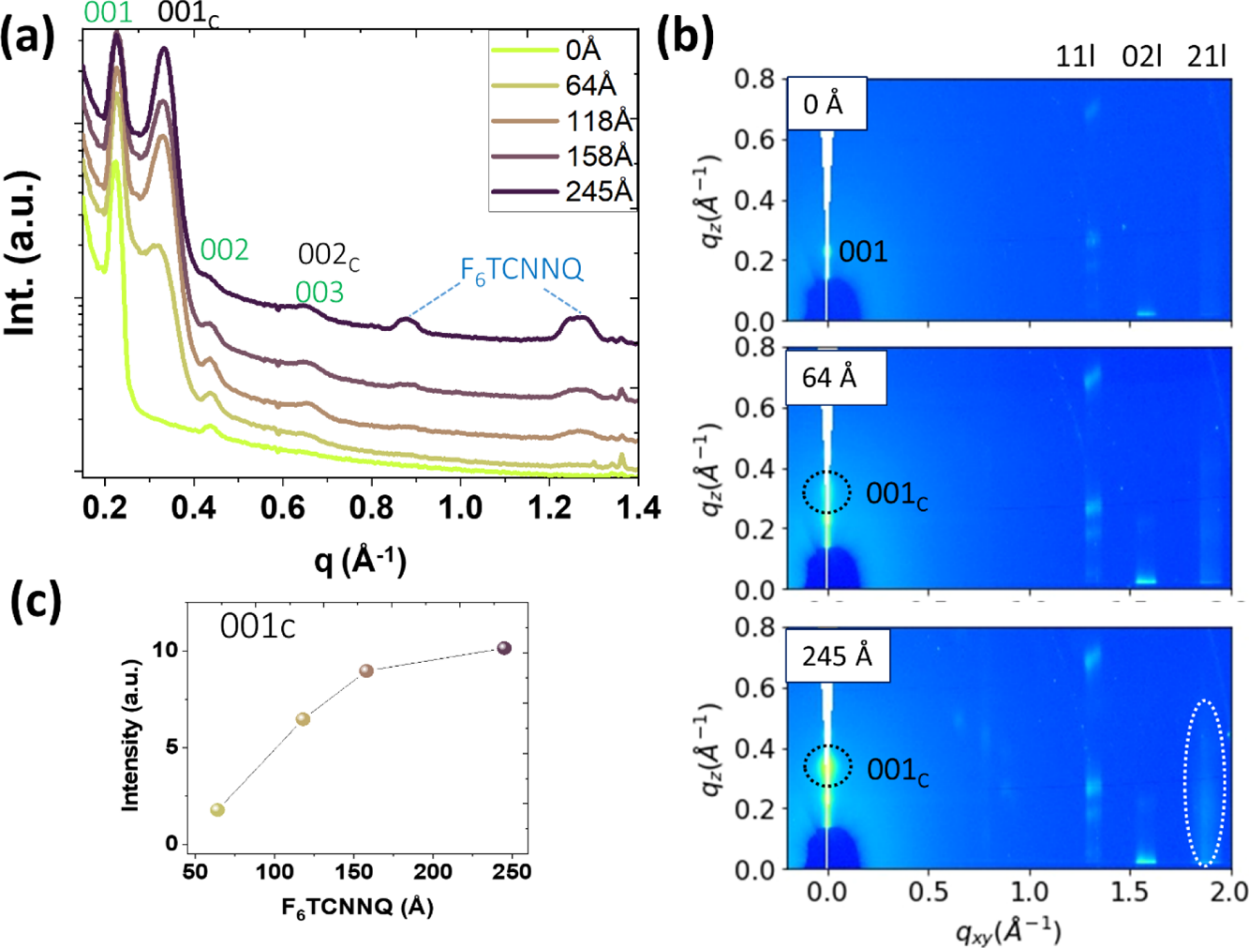
(a) OOP section cuts obtained from 2D GIWAXS images acquired *in situ* during the sequential deposition on top of 30 nm-thick C_8_-BTBT film (incident angle 4). The peak emerging at *q*_*z*_ = 0.33 Å^–1^ is labeled as (001)_C_ and corresponds to the formation of C_8_-BTBT/F_6_TCNNQ cocrystals. (b) Selected 2D GIWAXS maps for three stages of the F_6_TCNNQ deposition (0, 65, and 245 Å). (c) Intensity of the (001)_C_ peak as a function of the F_6_TCNNQ coverage.

At this point, knowing the details of the structural characteristics becomes relevant to understand the doping mechanism. To monitor any possible structural change derived from the incorporation of the dopant molecules at the OSC surface, we performed real-time GIWAXS measurements during the *in situ* deposition of F_6_TCNNQ onto a 30 nm-thick C_8_-BTBT film. The substrates used were of the same type as for the OFET fabrication. To avoid the influence of radiation damage during data acquisition, the sample was laterally displaced stepwise for data collection in fresh locations. Figure 4a displays the OOP section cuts acquired for the indicated layer thicknesses of the deposited F_6_TCNNQ (up to 245 Å); Figure 4b shows the 2D GIWAXS patterns for the C_8_-BTBT film before and after two depositions of F_6_TCNNQ, 65 and 245 Å, respectively. The pristine film exhibits the structural signature of the C_8_-BTBT thin film packing, with the (00*1*) Bragg reflection at *q*_*z*_ = 0.21 Å^–1^ and faint but measurable intensities at *q*_*xy*_ ≈ 1.31 Å^–1^, *q*_*xy*_ ≈ 1.57 Å^–1^, and *q*_*xy*_ ≈ 1.88 Å^–1^, respectively, assigned to the (11l), (02l), and (12l) diffraction rods. The deposition of F_6_TCNNQ leads to additional diffraction features attributed to the cocrystal formation. In addition to the (001)_C_ peak at *q*_*z*_ = 0.33 Å^–1^ (dotted circle), an increase of intensity is observed at *q*_*xy*_ = 1.86 Å^–1^ (enclosed by a dotted ellipse in the bottom panel of Figure 4b). This in-plane peak corresponds to a spacing of 3.4 Å in the real space and is ascribed to the π–π stacking of adjacent F_6_TCNNQ and C_8_-BTBT molecules in the cocrystal (in-plane section cuts are in Figure S7a). Although faint, another in-plane peak can be noticed at *q*_*xy*_ = 0.79 Å^–1^ (7.95 Å in real space), with a plausible correspondence to the other in-plane direction in the reciprocal space (i.e., edge-to-edge stacking of the aromatic cores). The absence of other diffraction features hinders a proper determination of the cocrystal structure. Figure 4c displays the (001)_C_ peak intensity as a function of F_6_TCNNQ deposition. This signal grows steadily from the early stage of F_6_TCNNQ deposition and slows down above approximately 150 Å. This evolution is accompanied by a peak narrowing, indicating the vertical growth of the cocrystals. The mean value of the vertical coherence length (CL) estimated from the full-width half-maximum of the (001)_C_ peak is CL ∼17.7 nm for the maximum amount of deposited F_6_TCNNQ (245 Å).

**Figure 5:**
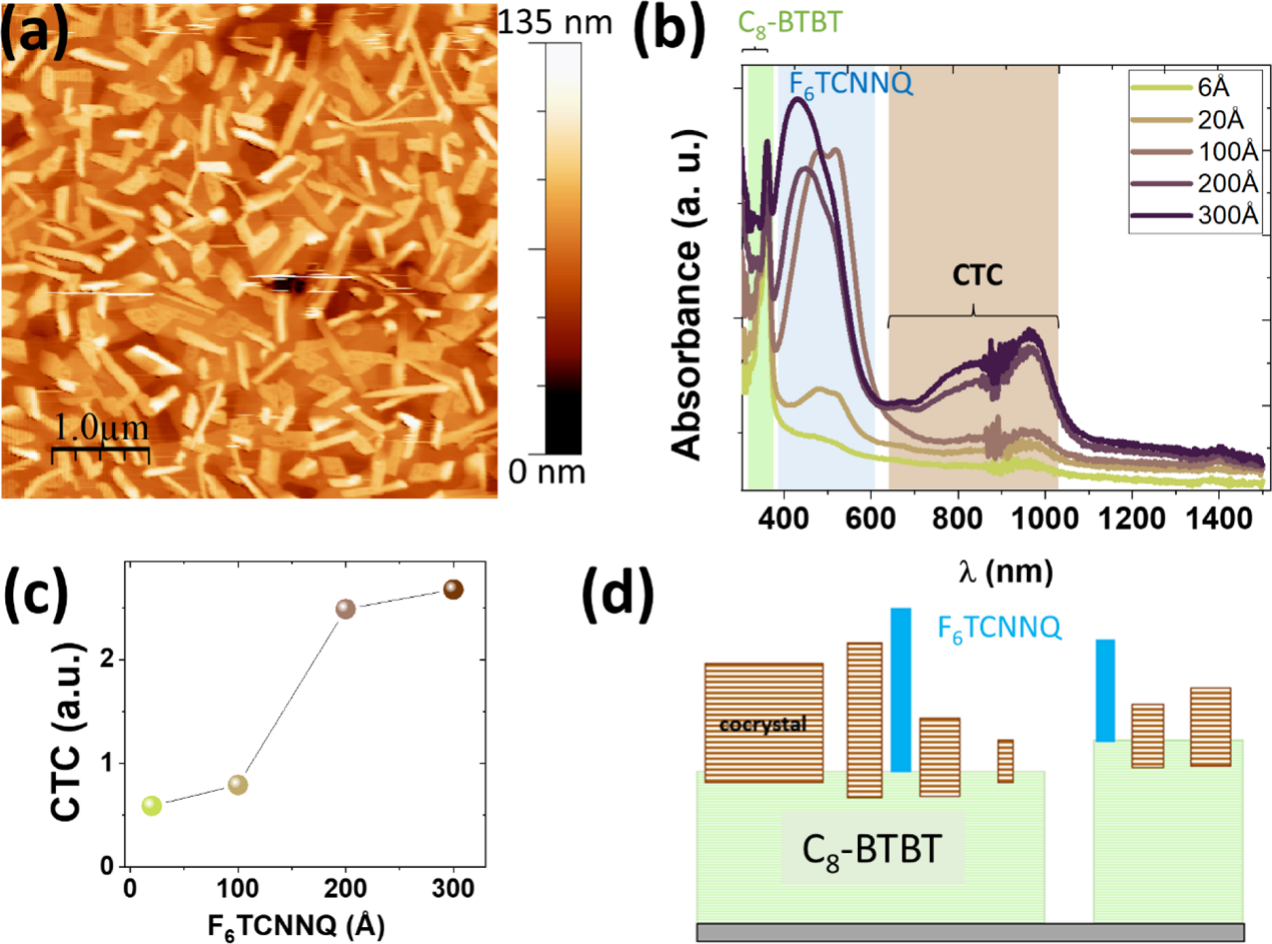
(a) Topographic image of the film surface after depositing 245 Å of F_6_TCNNQ on the C_8_-BTBT film. (b) UV–vis absorption spectra for the indicated F_6_TCNNQ layer thickness on top of C_8_-BTBT films. Colored regions correspond to the absorption ranges of the different molecular species (C_8_-BTBT, F_6_TCNNQ, and CTC cocrystal). (c) Area under the spectral region of the CTC as a function of F_6_TCNNQ coverage. (d) Cartoon illustrating the cocrystal formation.

It is also seen in Figure 4a for large F_6_TCNNQ coverages (>150 Å) the emergence of new structural features at *q*_*z*_ = 0.87 Å^–1^ and *q*_*z*_ = 1.26 Å^–1^, assigned to the crystallization of unmixed F_6_TCNNQ.^33,40^ The present results demonstrate the formation of cocrystals on the C_8_-BTBT surface, followed in a later stage by the crystallization of accumulated and unreacted F_6_TCNNQ molecules. The intensity evolution of the F_6_TCNNQ peak at *q*_*z*_ = 1.26 Å^–1^ is displayed in Figure S6. Complementary GIWAXS measurements acquired at an incident angle of 0.10° (i.e., below the critical angle of the film) show a higher intensity ratio of the cocrystal/C_8_-BTBT peak (for the first order diffraction) than for an angle of 0.15°, which supports the predominance of the cocrystal structure at the surface of the film (Figure S7b). It is now of interest to describe the film morphology as examined by AFM after the GIWAXS experiments. As it can be seen in Figure 5a, on top of a flat terraced surface, topographic images reveal the formation of rectangular crystallites with relatively high aspect ratios and flat-top facets with a height distribution centered around 20 and 50 nm. The lower structures plausibly correspond to the detected cocrystallites, in agreement with the vertical CL extracted from GIWAXS data. The steady growth of the cocrystals up to such a vertical size is quite remarkable. Since during the first stages of cocrystal formation at the interface the F_6_TCNNQ necessarily displaces part of the C_8_-BTBT molecules, the latter would remain on the surface ready to react with newly impinging F_6_TCNNQ. The number of C_8_-BTBT molecules available at each stage on the surface would depend on the diffusion and mass transport properties of the system, but it surely decreases with deposition time, and eventually, the reaction will stop. From that moment on, the excess of F_6_TCNNQ will nucleate to form pure F_6_TCNNQ aggregates (see below).

The CTC character of the cocrystals has been explored by performing UV–vis spectroscopy experiments for twin samples, prepared on glass simultaneously with the OFET fabrication. As it can be seen in Figure 5b, the formation of the CTC is supported by the emergence of the subgap absorption bands at 646 and 960 nm and their increase as a function of F_6_TCNNQ coverage. Note that the intensity of the peak centered at around 960 nm (Figure 5c) follows a similar trend as the intensity of the cocrystal (001)_C_ Bragg peak (Figure 4c). Interestingly, the absorption band of neutral F_6_TCNNQ is already observed for low coverage, although at that stage, the dopant is undetected by GIWAXS. The large intensity of this band for increasing coverage agrees with the presence of the quite tall (∼50 nm) aggregates of F_6_TCNNQ in the AFM image and the appearance of F_6_TCNNQ structural features observed in GIWAXS (Figure 4a).

In summary, the electrical characteristics of the OFETs, the evolution of the film structure, and the optical absorption during the incorporation of the dopant are fully consistent and demonstrate that the development of CTC cocrystals at the C_8_-BTBT surface drives the p-type doping of the C_8_-BTBT film. As the CTC cocrystals grow, the number of mobile charge carriers in the channel increases, causing *V*_th_ to shift and *I*_off_ to increase. Outstandingly, and conversely, to bulk doping by codeposition where cocrystals are embedded in the C_8_-BTBT film, the cocrystals evolve from the film surface, thus leaving the microstructure of the underlying film unperturbed and leading to an improvement of the field-effect mobility of increasingly doped OFETs. For a sufficiently large coverage (>100 Å of F_6_TCNNQ in the present case), the decrease of μ may be due to the large vertical size of the crystallites (F_6_TCNNQ and cocrystals), having a detrimental effect on the electrode contacts for the staggered bottom-gate device geometry of the studied OFETs.

### Morphology and Electronic Properties of the Doped Interface

**Figure 6:**
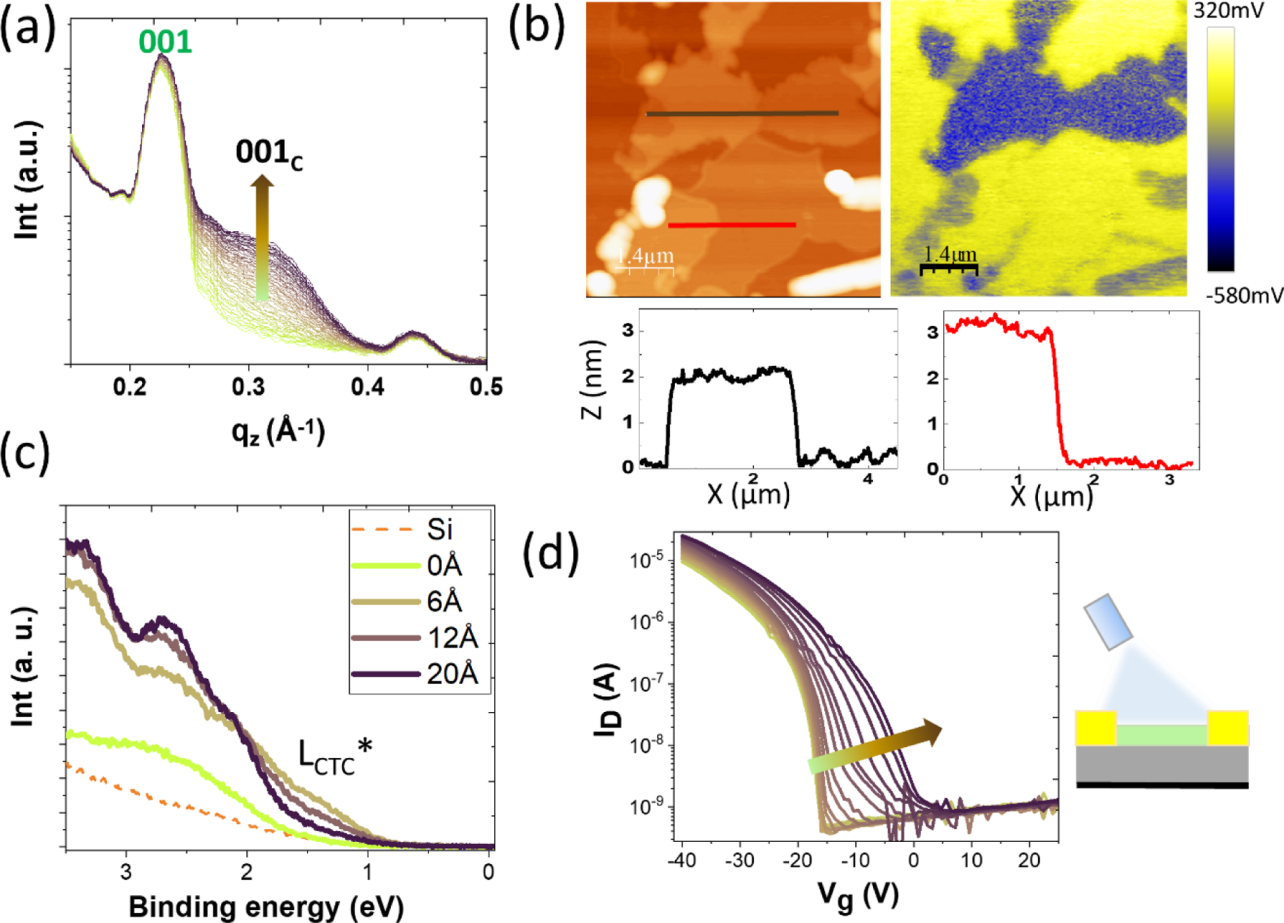
(a) OOP section cut profiles extracted from the 2D GIWAXS images acquired in real time during deposition of F_6_TCNNQ (up to 35 Å) on top of a 30 nm-thick C_8_-BTBT film. The incident angle is 0.11°. (b) Topography (left) and SP map (right) measured by KPFM for F_6_TCNNQ (15 Å) on top of a 15 nm-thick C_8_-BTBT thin film. Dark (low SP) and bright (high SP) patches correspond to the cocrystal and bare C_8_-BTBT surfaces, respectively. Bottom: height profiles along the segments indicated in the topographic image. (c) VB measured by UPS for the C_8_-BTBT films after the indicated depositions of F_6_TCNNQ. The spectra for the clean substrate are also shown. (d) Transfer curves (*V*_DS_ = −5 V) acquired *in situ* during the deposition of F_6_TCNNQ on top of a 15 nm-thick C_8_-BTBT film. Each curve corresponds to an increase of 1 Å F_6_TCNNQ up to 55 Å. The cartoon illustrates the OFET configuration.

A deep understanding of the cocrystals’ growth mechanism is beyond the scope of this work and needs further studies. However, in the present context, it is possible to clarify some interfacial details, both structural and electronic, associated with the early stage of cocrystal growth. To that end, Figure 6a displays the evolution of the structure monitored in real time from the initial stage of the deposition of F_6_TCNNQ up to a nominal thickness of 35 Å. The progressive increase of the scattering signal around *q*_*z*_ = 0.33 Å^–1^ for this coverage range is indicative of initial formation and growth of the cocrystal that gives rise to the (001)_C_ peak for ulterior depositions (see Figure 4). The large width of the incipient (001)_C_ peak is related to a small number of ordered planes in the cocrystal from which X-rays are coherently scattered (indicating, in other words, the small vertical size of the cocrystal structure). In addition, notable information comes out from the analysis of the AFM data obtained at the very beginning of the dopant deposition. Figure 6b shows the morphology of a 15 nm-thick C_8_-BTBT film after the deposition of 15 Å of F_6_TCNNQ and the surface potential (SP) map simultaneously acquired by means of KPFM. As described in the Experimental Methods section for the setup employed, higher (lower) SP corresponds to lower (higher) surface work function (ϕ). Interestingly, the contrast in the SP map indicates the presence of surface regions with considerably different ϕ (Δϕ ≈ 0.35 eV). Terraces located in the bottom part of the image have the expected height of C_8_-BTBT (∼3 nm) (see red profile), whereas the terrace height at the larger ϕ region (upper part) is ∼2 nm (black profile), in excellent agreement with the interlayer spacing in the lamellar structure of the cocrystal. Thus, the GIWAXS and AFM data give evidence of a two-dimensional assembly of the mixed cocrystal on the surface of C_8_-BTBT at the early growth stage and a stacked growth of F_6_TCNNQ with increasing coverage.

The valence band (VB) measured by UPS, shown in Figure 6c for pristine C_8_-BTBT and selected dopant coverages of F_6_TCNNQ, reveals a clear increase of the density of states (DOS) at low binding energies (with an energy onset at BE ≈ 0.7 eV) upon the deposition of 6 Å of F_6_TCNNQ, which can be assigned to the partially filled LUMO of the CTC. This fact can be interpreted as evidence of charge transfer between the OSC and the CTC. Remarkably, this electronic feature is a purely interfacial DOS since its intensity is attenuated with further F_6_TCNNQ coverage. Conversely, the DOS above 2 V increases with coverage, which plausibly arises from the occupied molecular orbitals of the CTC (HOMO_CTC_). UPS data also show an increase in work function (see Figure S8), demonstrating that the ionization potential of the CTC is substantially higher than that of C_8_-BTBT.

Finally, the change in the electrical characteristics of C_8_-BTBT OFETs was *in situ*-evaluated during the deposition of F_6_TCNNQ. In this case, we employed a coplanar bottom-gate geometry with 200 nm SiO_2_ as gate dielectric (further details in the Supporting Information). Figure 6d shows the evolution of the transfer curves during the deposition of F_6_TCNNQ (for coverages up to 55 Å). For pristine C_8_-BTBT, a negative *V*_on_ is observed due to the presence of negative traps at the dielectric–OSC interface. As the cocrystal forms by increasing deposition of F_6_TCNNQ, the transfer curves experience a shift of *V*_th_ to a lesser negative gate value so that the on voltage goes from *V*_on_ = −16 to 5 V without substantial effect on the *I*_off_ current (*V*_th_ shifts from −13.1 to −5.6 V). This fact demonstrates that surface doping by CTC in the low-coverage regime may be used to passivate traps and reduce the *V*_th_.

As a final note, we emphasize that the additional advantage of the surface doping introduced here, as compared to bulk doping, is that the quality of the OSC thin film is preserved independently of the amount of the dopant. While in coevaporation, an excess of F_6_TCNNQ dramatically disturbs the quality of the OSC film, an excess of dopant in the postdeposition approach results in the nucleation of F_6_TCNNQ crystals at the surface that do not alter the device properties.

## Conclusions

This work demonstrates that surface doping can take place via CTC formation, shown here for the OSC C_8_-BTBT and F_6_TCNNQ as a p-type dopant. The key finding of this work is proof of the superior suitability of CTC surface doping for the purpose of improving the field-effect mobility and reducing the *V*_th_ in OFETs, as well as increasing the density of free carriers in the OSC film. The combined analysis by AFM, GIWAXS, and UV–vis absorption consistently reveals the formation of a cocrystal structure at the surface of the C_8_-BTBT film that grows with the increasing amount of deposited F_6_TCNNQ. The observation of interfacial gap states by UPS provides evidence of electron charge transfer at the interface. Importantly, although CTC formation and its capability for generating mobile charges are demonstrated to occur in both approaches, coevaporation and sequential deposition of OSCs and the dopant, the different spatial distribution of the cocrystals critically determines the field-effect mobility of the final devices. Whereas the structural order of the OSC thin film remains unaffected independently of the amount of the deposited dopant for the sequential deposition of F_6_TCNNQ, the codeposition of both molecules leads to a decrease of the field-effect mobility even for a dopant ratio of about 1 mol % due to a reduction of the lateral size of the grain morphology.

Insight acquired into the interface properties reveals the 2D assembly of the cocrystal at the C_8_-BTBT surface at the early stage of F_6_TCNNQ deposition with the formation of a new interfacial DOS signal attributed to the CTC-filled LUMO due to charge transfer across the interface.

In short, CTC surface doping is an advantageous strategy to improve the OFET parameters, given that it preserves the thin-film structure integrity regardless of the amount of the dopant. We believe that the conclusions of this work can be of general applicability to simplify the optimization of OSC/dopant ratios during the fabrication of organic electronic-doped devices.

## Supporting Information Available

Output curves of the OFETs, mobility calculated for different channel lengths, comparison for two sets of OFETs fabricated either with the gold contacts placed on top of the C_8_-BTBT film prior to F_6_TCNNQ or with F_6_TCNNQ deposited over the entire surface, additional 2D GIWAXS data, GIWAXS data for two incident angles of 0.10 and 0.15°, and plot with the secondary electron cutoff region measured by UPS (PDF)
