# Supplementary material for: Charge-Transfer Complexes in Organic Field-Effect Transistors: Superior Suitability for Surface Doping
Source: ACS Appl Mater Interfaces. 2022 Sep 20;14(39):44632–41. doi: 10.1021/acsami.2c09168 (PMC9542699; doi:10.1021/acsami.2c09168)
Supplement: Supplementary file 1 — am2c09168_si_001.pdf [file am2c09168_si_001.pdf]

Supporting Information

Charge Transfer Complexes in Organic Field-Effect Transistors: Superior Suitability for Surface Doping

Adara Babuji,[1] Alba Cazorla,[1] Eduardo Solano,[3] Carsten Habenicht,[2] Hans Kleemann,[2] Carmen Ocal,[1] Karl Leo,[2] Esther Barrena[1]**

[1] Institut de Ciència de Materials de Barcelona (ICMAB), Campus de la UAB, Bellaterra, Barcelona, 08193, Spain

[2] Dresden Integrated Center for Applied Physics and Photonic Materials (IAPP), Dresden, 01062, Germany

[3]ALBA synchrotron, C/ de la Llum 2-26. Cerdanyola del Vallès, Barcelona, 08290, Spain

*ebarrena@icmab.es, *karl.leo@tu-dresden.de

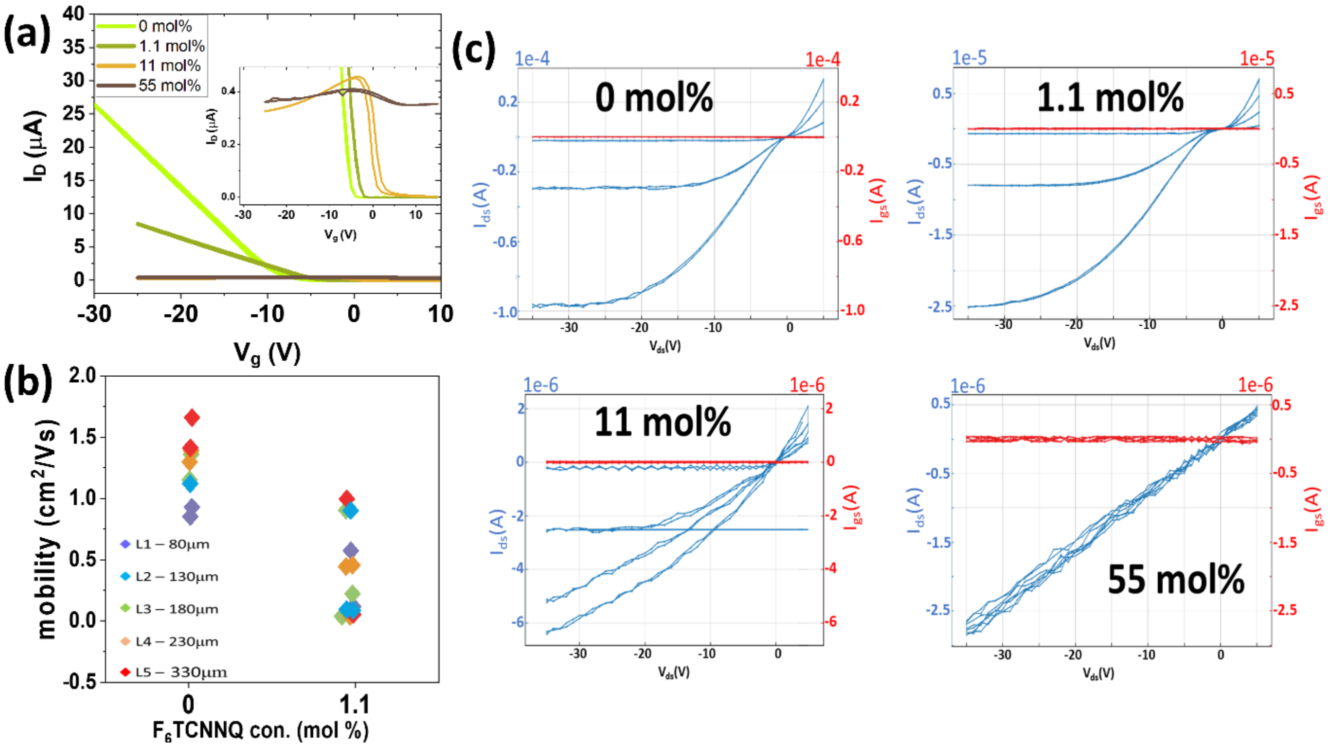

Figure S1. Co-evaporated thin films: (a) Linear transfer curves in linear scale at V_D= –5 V of the OFETs for co-evaporated thin films (L = 230 μm) for the indicated doing concentrations of F_6TCNNQ. (b) Mobility calculated for different channel lengths. (c) Output curves (for Vg = 0 V, -10 V, -20 V, -30 V).

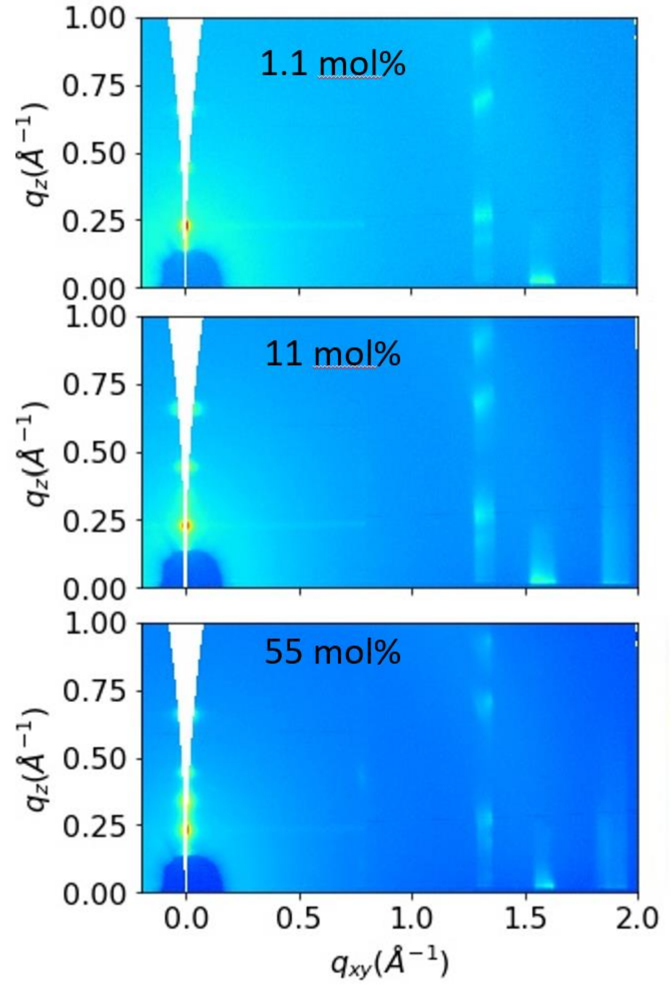

Figure S2. Co-evaporated thin films: GIWAXS 2D maps obtained for doped co-evaporated thin films with the indicated doping concentrations.

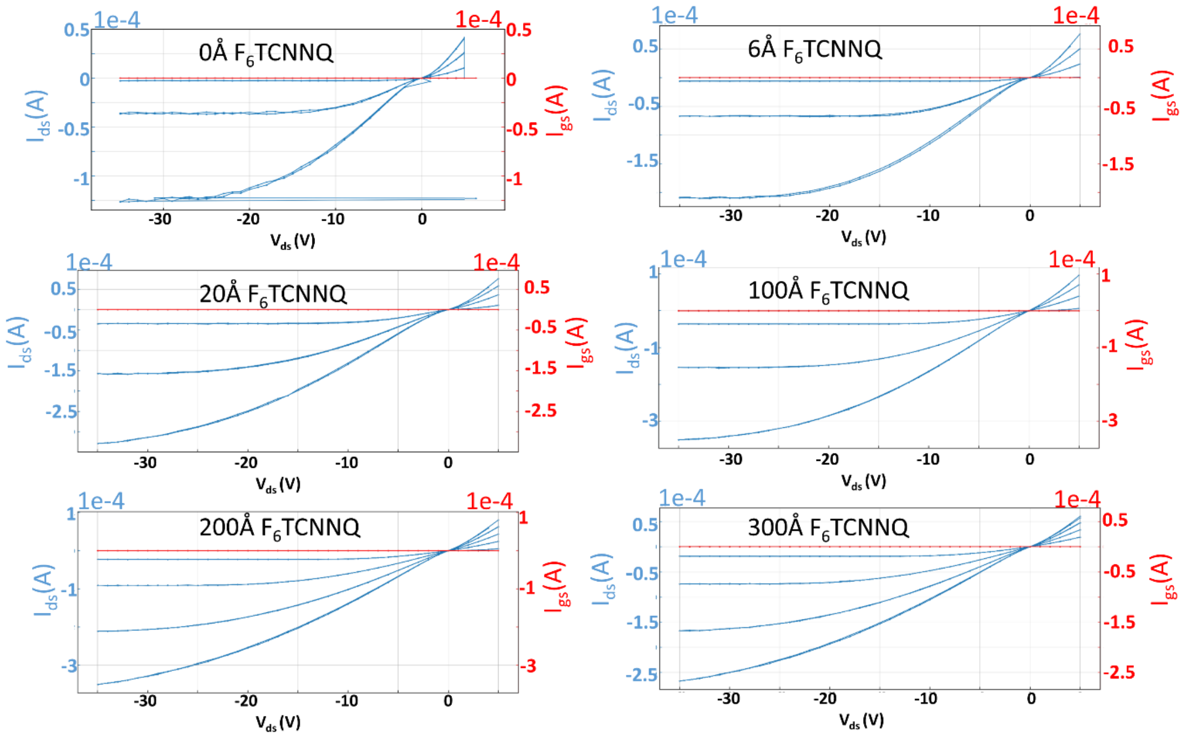

Figure S3. Sequential deposition of C_8-BTBT and F_6TCNNQ: Output curves (for Vg = 0 V, -10 V, -20 V, -30 V) of C_8-BTBT OFETs (L=180 µm) for different coverages (indicated in the graph) of F_6TCNNQ deposited over the C_8-BTBT film.

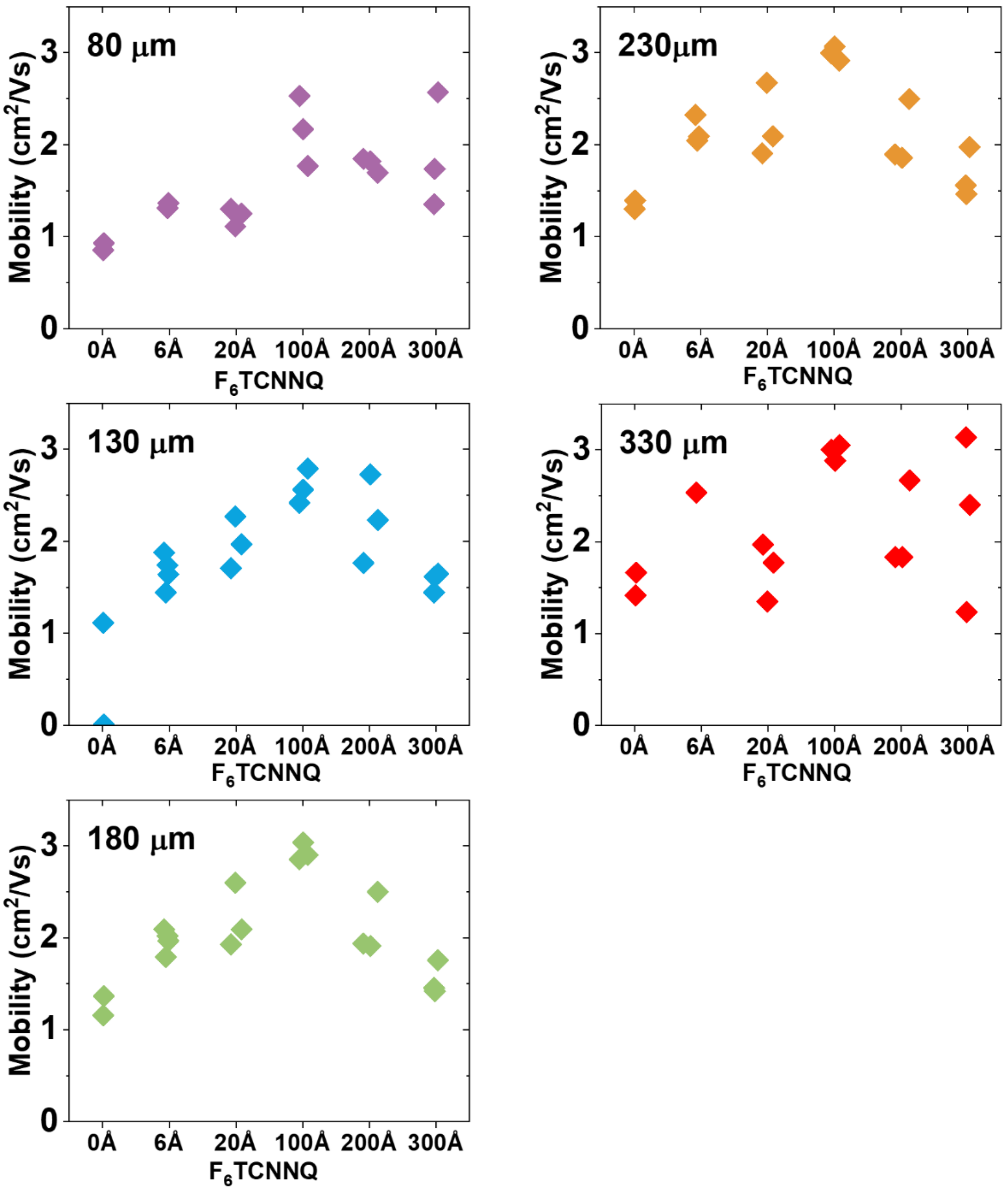

Figure S4. Sequential deposition of C_8-BTBT and F_6TCNNQ: Mobility calculated for different channel length as a function of the F_6TCNNQ coverage.

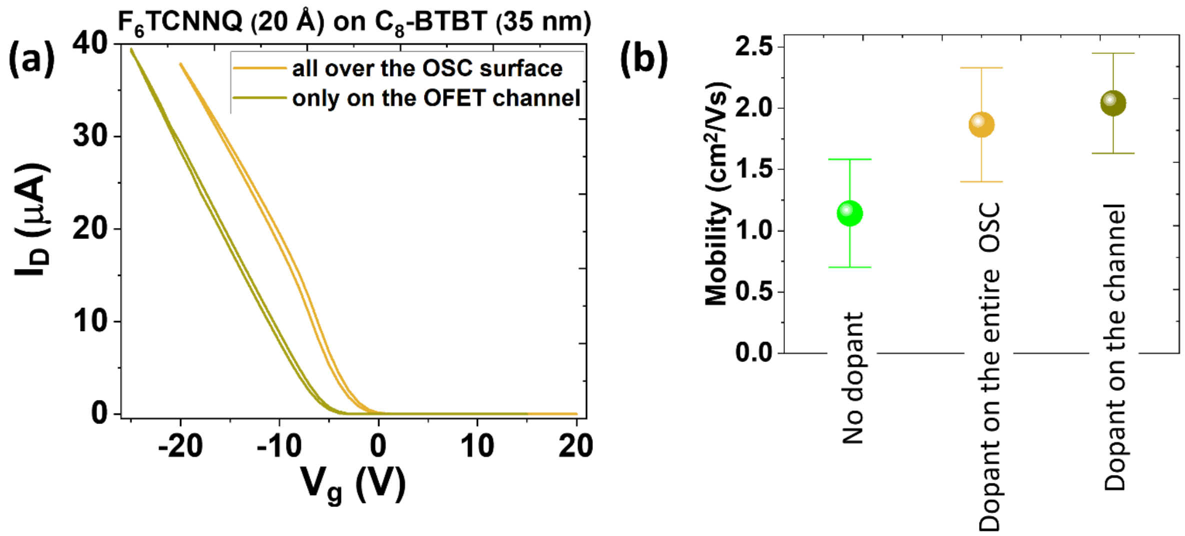

Figure S5. Comparison for two set of OFETs fabricated either with the gold contacts placed on top of the C_8-BTBT film prior to F_6TCNNQ deposition, to guarantee that the dopant molecules were only on the channel region, or with F_6TCNNQ deposited over the entire surface, also under the contacts. (a) Transfer curves at V_{ds} = -5 V (L= 230 µm). (b) Measured linear mobility for 15 devices of each type. The error bars are the data standard deviation. The light green data in (b) corresponds to the pristine devices.

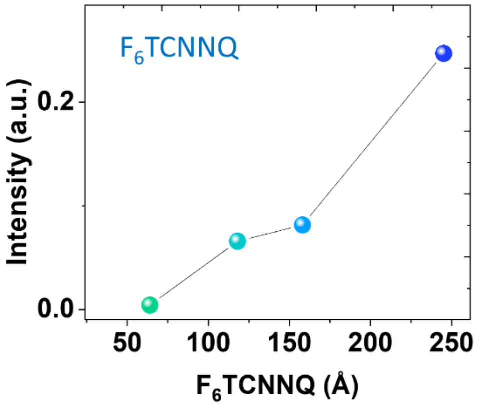

Figure S6. Sequential deposition of C_8-BTBT and F_6TCNNQ. Evolution of the integrated intensity of F_6TCNNQ peak at q = 1.26 $Å^{-1}$ in the OOP section cuts obtained from 2D GIWAXS images acquired in-situ during the sequential deposition on top of C_8-BTBT film (incident angle of 0.10°).

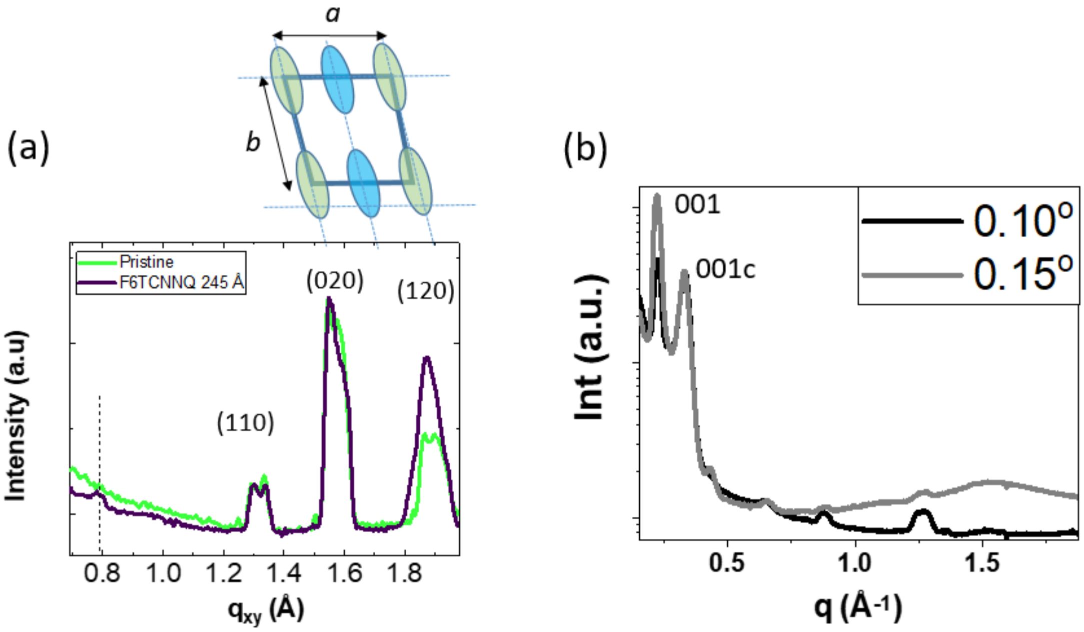

Figure S7. (a) Top: In-plane unit cell of the co-crystalline structure formed by F6TCNNQ and C_8-BTBT with the schematic representation of the stacking of their conjugated cores. Bottom: Section cuts from the GIWAXS data for the pristine film and after the deposition of 245 Å of F_6TCNNQ. The intensity has been integrated in a vertical slab of ≈ 0.2 $Å^{-1}$ along q_z and plotted as a function of q_{xy}. The deposition of F_6TCNNQ leads to an increase of the in-plane intensity at q_{xy} = 1.86 $Å^{-1}$ (spacing of 3.4 Å), overlapping with the *(21l)* rod of C_8-BTBT. In addition, a new peak is visible at q_{xy} = 0.79 $Å^{-1}$ (spacing of 7.95 Å), marked with a dashed vertical line. These two peaks, observed at q_{xy} = 1.86 $Å^{-1}$ and 0.79 $Å^{-1}$, have a plausible correspondence with the diffraction from *(200)* (π-stacking along the a-axis) and *(010)* planes in the co-crystal, respectively. (b) The OOP section cuts obtained from the GIWAXS data for two incident angles of 0.10° (surface sensitive) and 0.15° (probing the whole film thickness) for 245 Å of F_6TCNNQ deposited on C_8-BTBT (30 nm thick). The scattering intensity observed around q = 1.5 $Å^{-1}$ observed for an incident angle of 0.15° arises from the underlying CYTOP film. These data show the predominance of the co-crystal structure at the surface of the film.

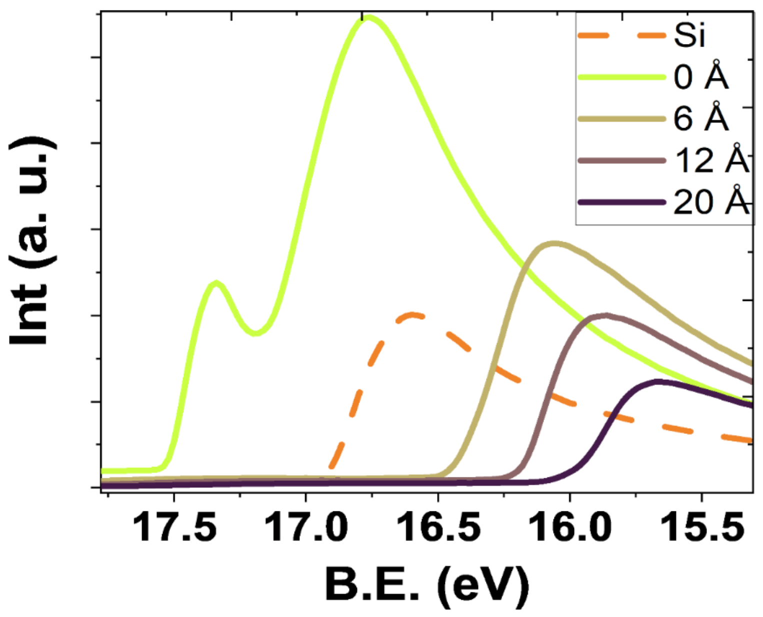

Figure S8. Secondary Electron Cut off region (SECO) measured by UPS for the C_8-BTBT films after the indicated depositions of F_6TCNNQ.
